# Immunological mechanism behind reactivated cryptococcosis in persistently infected mice following FTY720 treatment

**DOI:** 10.1128/iai.00612-25

**Published:** 2026-04-30

**Authors:** Michiko Yoshida, Ko Sato, Nana Nakahata, Hayato Sato, Reina Ohagi, Emi Kanno, Hiromasa Tanno, Keiko Ishii, Tetsuji Aoyagi, Atsuo Kikuchi, Kazuyoshi Kawakami

**Affiliations:** 1 Department of Pediatrics, Tohoku University Graduate School of Medicine 38047, Sendai, Miyagi, Japan; 2 Department of Clinical Infectious Diseases, Tohoku University Graduate School of Medicine 38047, Sendai, Miyagi, Japan; 3 Department of Clinical Microbiology and Infection, Tohoku University Graduate School of Medicine 38047, Sendai, Miyagi, Japan; 4 Department of Translational Science for Nursing, Tohoku University Graduate School of Medicine 38047, Sendai, Miyagi, Japan; 5 Hirose Hospital 690907, Sendai, Miyagi, Japan; Rutgers New Jersey Medical School Division of Infectious Diseases, Newark, New Jersey, USA

**Keywords:** FTY720, reactivation, latent infection, cryptococcosis

## Abstract

The *Cryptococcus neoformans* species complex (CNSC), responsible for cryptococcosis, is controlled by Th1-type immunity, in which IFN-γ-activated macrophages form granulomas that contain infection. Nevertheless, CNSC can evade host immunity and, after the primary infection phase, can shift to a latent infection similar to tuberculosis. Reactivation is thought to occur when immune control fails, but the underlying mechanisms remain poorly understood. FTY720, a functional antagonist of sphingosine-1-phosphate receptors, is primarily used in the treatment of multiple sclerosis. However, there have been reports linking its use to instances of cryptococcosis in patients undergoing treatment. To explore this, we established a novel mouse model using CnT-II mice, which express CD4^+^ T cell receptors specific for chitin deacetylase 2 (Cda2), a major T cell antigen of CNSC. Following infection with encapsulated CNSC, mice developed persistent pulmonary fungal burdens (10²–10³ CFU) accompanied by granulomatous responses, modeling latent infection. Upon FTY720 administration, mice exhibited increased pulmonary fungal burdens, disrupted granuloma integrity, and reduced IFN-γ and IL-12 production. FTY720 also markedly decreased CD4^+^ effector memory (Tem) and effector T cells (Teff) in the lungs, with a particularly profound loss of IFN-γ-producing Tem cells. These findings indicate that our model successfully recapitulates latent cryptococcal infection and reactivation, and demonstrate that FTY720 promotes relapse by depleting protective IFN-γ-producing CD4^+^ Tem cells and impairing Th1-mediated immunity. Given the rising use of FTY720, our study highlights its potential risk in patients with subclinical cryptococcosis and underscores the need for preventive strategies to avert disease progression.

## INTRODUCTION

*Cryptococcus* spp. is a yeast-like fungus commonly found in the environment, particularly in soil, and typically infects hosts via the airborne route (1). The main pathogenic species are part of the *Cryptococcus neoformans* species complex (CNSC), including *C. neoformans* and *C. deneoformans* (formerly known as *C. neoformans* var. *neoformans*, serotype D) (2). The lungs are the primary site of cryptococcosis, which can disseminate to other organs, including the central nervous system (2). While pulmonary cryptococcosis is usually asymptomatic in immunocompetent hosts, immunocompromised hosts may develop severe conditions like meningoencephalitis (3). Once primarily associated with AIDS, cryptococcosis is now increasingly observed in patients with other forms of immunodeficiency, particularly in developed countries (4, 5). In 2022, the World Health Organization ranked CNSC as the top pathogen in its first fungal priority pathogens list due to its significant impact on society (6).

Understanding the immune response to CNSC is crucial for addressing the threat of cryptococcosis. This fungus is a facultative intracellular pathogen that can persist within macrophages after phagocytosis (7). The elimination of these pathogens is tightly regulated by type 1 helper T (Th1) immune responses during the primary infection phase (8). Upon CNSC infection, dendritic cells recognize the fungus, migrate to the draining lymph nodes, and present fungal antigens to CD4^+^-naïve T cells (Tnaïve), leading to the development of Th1 effector T cells (Teff). Th1 cells then return to the infection site, where antigen-presenting cells (APCs) stimulate IFN-γ secretion, activating macrophages to form granulomas and contain the fungus (9–11).

Clinical observations suggest that cryptococcal infection may enter a latent phase after the initial immune response, similar to tuberculosis (12, 13). Under immunosuppressive conditions, this latent cryptococcal infection could reactivate due to disruption of the antigen-specific T-cell response, though the exact mechanism remains unclear. We hypothesize that some Th1 cells differentiate into memory T cells (Tm) after the acute infection phase, and IFN-γ production from antigen-specific Tm cells helps maintain granulomas and suppresses disease reactivation. To explore this possibility, an analysis of the immune response in the lungs using an appropriate animal model is required.

Although several mouse models of latent cryptococcal infection have been reported (14, 15), two major issues remain. First, these models employed capsular mutant or attenuated strains, as wild-type mice infected with capsular strains typically succumb to the infection and cannot survive for a prolonged period (14). However, since the capsule is a crucial virulence factor for this fungus, enabling it to evade the immune system (16), the use of capsular mutant or attenuated strains may not accurately reflect natural infection. Second, antigen-nonspecific Tm cells, such as memory phenotype T cells (MPTs), can complicate the analysis of cryptococcus-specific Tm cells. MPTs, which express the same surface marker as Tm cells, produce IFN-γ in response to cytokine stimulation, such as IL-12, without requiring antigen presence (17). We previously reported the presence of MPTs in the lungs during the early phase of cryptococcal infection (18). To address these issues, we established a transgenic mouse model, CnT-II, which expresses high levels of cryptococcal antigen chitin deacetylase 2 (Cda2)-specific T cell receptors, with approximately 90% of CD4^+^ T cells being Cda2-specific T cells (19).

Fingolimod (FTY720) is primarily phosphorylated by sphingosine kinase 2 (SphK2) to form FTY720 phosphate (FTY720P), which acts as a functional antagonist of sphingosine-1-phosphate (S1P) receptors (S1PR) (20). While FTY720 affects a variety of immune cells, its most well-characterized action is on T cells, where it induces internalization of S1P receptor 1 (S1PR1), thereby inhibiting their egress from lymphoid organs and migration to the central nervous system (20). FTY720 is used to treat multiple sclerosis (MS), an autoimmune disorder affecting the central nervous system. Recently, cases of cryptococcosis have been reported in patients treated with FTY720 (21, 22), while only a few cases of active tuberculosis have been seen (23, 24). In contrast, TNF-α inhibitors increase the risk of active tuberculosis but are not associated with cryptococcosis (25, 26). These observations suggest different mechanisms of susceptibility and a unique role for FTY720 in the onset of cryptococcosis.

In the present study, we aimed to establish a novel mouse model of latent cryptococcal infection, validate the onset of cryptococcal reactivation induced by FTY720, and investigate the underlying mechanisms using this model.

## RESULTS

### Establishment of a novel latent cryptococcal infection mouse model

**Fig 1:**
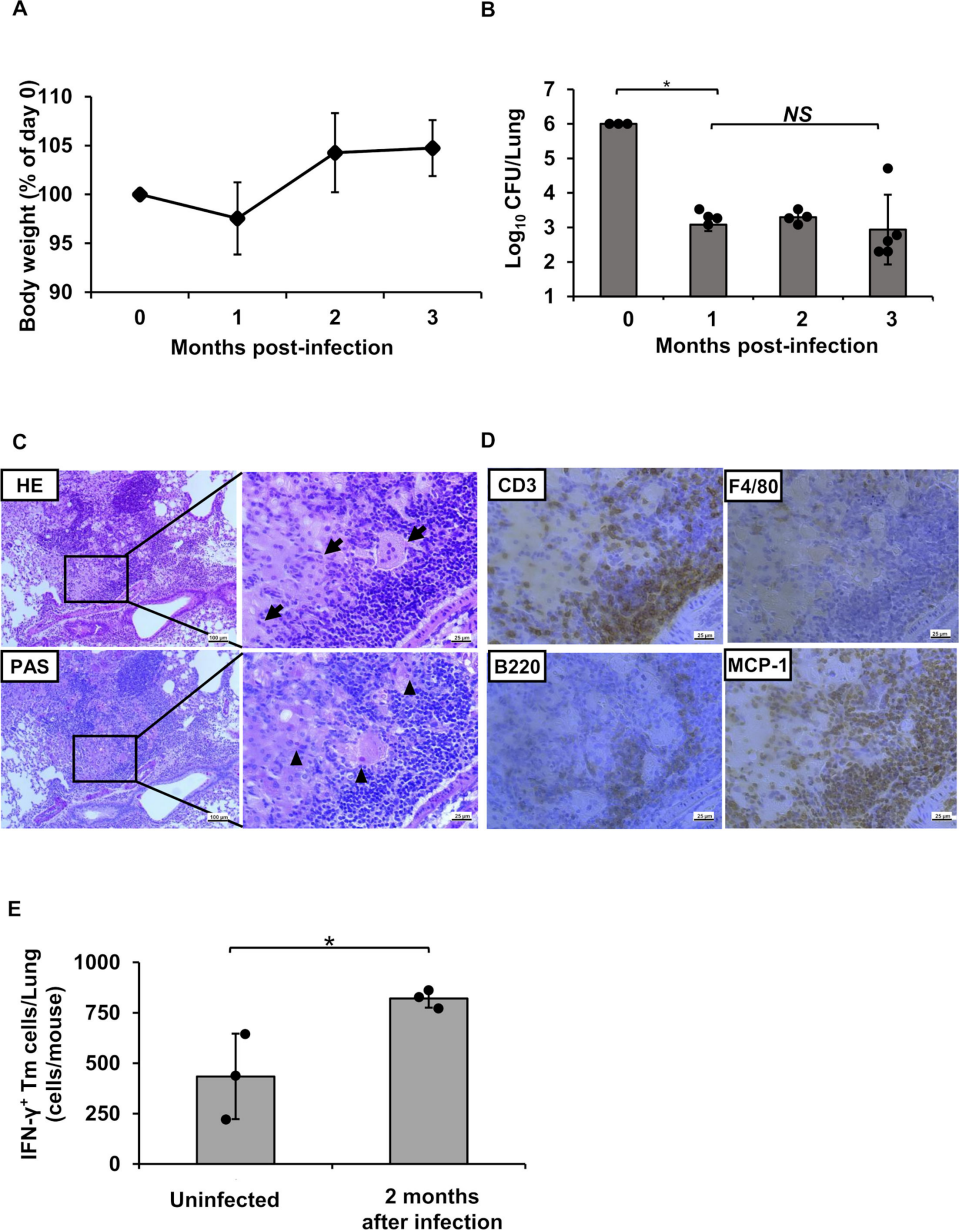
Establishment of a novel mouse model of latent cryptococcal infection. (**A**) Time course of body weight post-infection is shown (*n* = 3). (**B**) Time course of fungal burden in the lungs is shown (*n* = 3–5). (**C and D**) Sections of the lungs at 2 months post-infection were stained with hematoxylin-eosin (HE) and periodic acid-Schiff (PAS) and observed under a light microscope. Original magnification: left panels, ×10; right panels, ×40. Representative images are shown. Arrows indicate multinucleated giant cells, and triangles indicate fungal bodies. (**D**) Immunostained images within the granulomatous response of serial lung sections with anti-CD3, anti-B220, anti-F4/80, and anti-MCP-1 mAb. Original magnification: ×40. (**E**) The number of IFN-γ^+^ CD4^+^ memory T cells (Tm) in the lungs of uninfected mice and mice at 2 months post-infection was analyzed using flow cytometry (*n* = 3–4 in each group). Each symbol represents a separate mouse, and the bars indicate the mean ± SD. NS, not significant. *P < 0.05. Statistical analysis was performed using Welch’s *t*-test.

We aimed to establish a novel mouse model of latent cryptococcal infection using a capsular strain. In initial experiments, wild-type (WT) mice were infected with B3501. In these mice, fungal burdens in the lungs increased over time, and long-term survival was limited (Fig. S1), indicating that WT mice were unsuitable for modeling latent infection. Infection of CnT-II mice with the highly virulent strain H99 also resulted in lethal disease, precluding the establishment of a chronic or latent infection model. Based on these findings, we subsequently used CnT-II mice infected with B3501 to establish a latent infection model. Several criteria confirmed successful establishment of the model, including the absence of significant weight loss or clinical symptoms, persistent lung fungal presence, granuloma formation in the lungs (27), and CD4^+^ Tm cells in the lungs. Up to 3 months post-infection, mice showed stable body weight (Fig. 1A) and no clinical symptoms such as lethargy, piloerection, or rapid breathing. Fungal burdens in the lungs decreased to 10²−10³ CFU/lung after 1 month and remained stable until 3 months (Fig. 1B). No fungal spread to the spleen and brain was observed (data not shown). Histopathology revealed granulomatous structures composed of giant cells, macrophages, T cells, and B cells containing fungi. Monocyte chemoattractant protein-1 (MCP-1/CCL2), a chemokine that induces CD4^+^ T cells and monocyte/macrophage recruitment in the pulmonary cryptococcal infection model (28), showed consistent expression in T cells and B cells (Fig. 1C and D). Additionally, an increase in IFN-γ^+^ CD4^+^ Tm cells was detected in the lungs of infected mice (Fig. 1E). These results demonstrate that this model fulfills the criteria for latent cryptococcal infection. Because granulomatous structures had already formed by 2 months post-infection and the pulmonary fungal burden had stabilized thereafter, mice infected for at least 2 months were used as a model of latent cryptococcal infection in subsequent experiments.

### Reactivation of cryptococcal infection by FTY720 treatment

**Fig 2:**
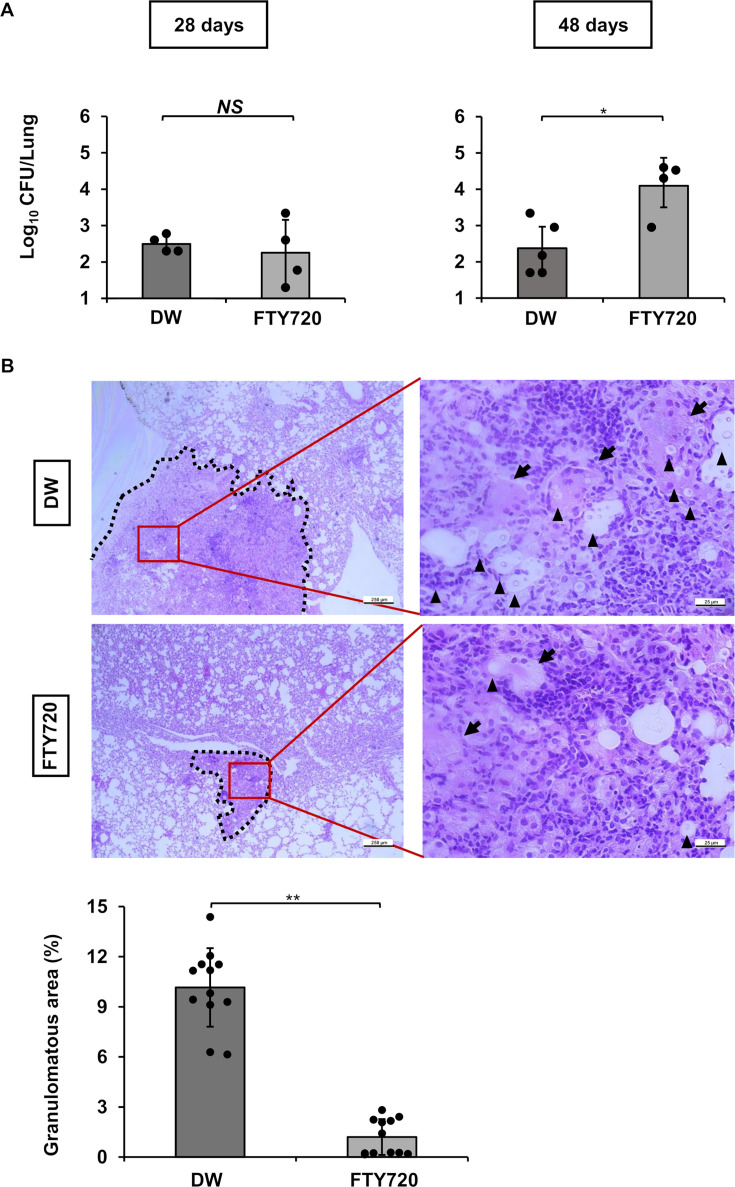
Reactivation of cryptococcosis induced by FTY720 treatment. Mice with latent cryptococcal infection were orally administered FTY720 daily for 28 and 48 days. Distilled water (DW) was used as the control. (**A**) The fungal burden in the lungs was assessed at 28 and 48 days post-treatment (*n* = 4–5 in each group). (**B**) Sections of the lungs at 28 days post-treatment were stained with HE and observed under a light microscope. Arrows indicate multinucleated giant cells, and triangles indicate fungal bodies. Representative pictures are shown. Original magnification: left panel, ×4; right panel, ×40. Granulomatous response (dotted lines) and its area were calculated (right panel) (*n* = 12 in each group). Data are expressed as the mean ± SD. Each symbol represents a separate section, and the bars indicate the mean ± SD. NS, not significant. *P < 0.05. **P < 0.01. Statistical analysis was performed using Welch’s *t*-test (Fig. 2B) and the Mann-Whitney U-test (Fig. 2B).

Using this model, we evaluated the potential of FTY720 to reactivate cryptococcal infection. Mice were treated with either FTY720 or DW, and the fungal burdens and granulomatous response in the lungs were assessed. The fungal burdens in the lungs were not significantly different between the two groups at 28 days post-treatment; however, they significantly increased in the FTY720-treated mice compared to controls at 48 days (Fig. 2A). FTY720-treated mice exhibited a significant reduction in granulomatous tissue compared to controls at 28 days post-treatment (Fig. 2B). These results suggest that FTY720 treatment may lead to the reactivation of cryptococcal infection.

### Effects of FTY720 treatment on Th1-related cytokine and chemokine production

**Fig 3:**
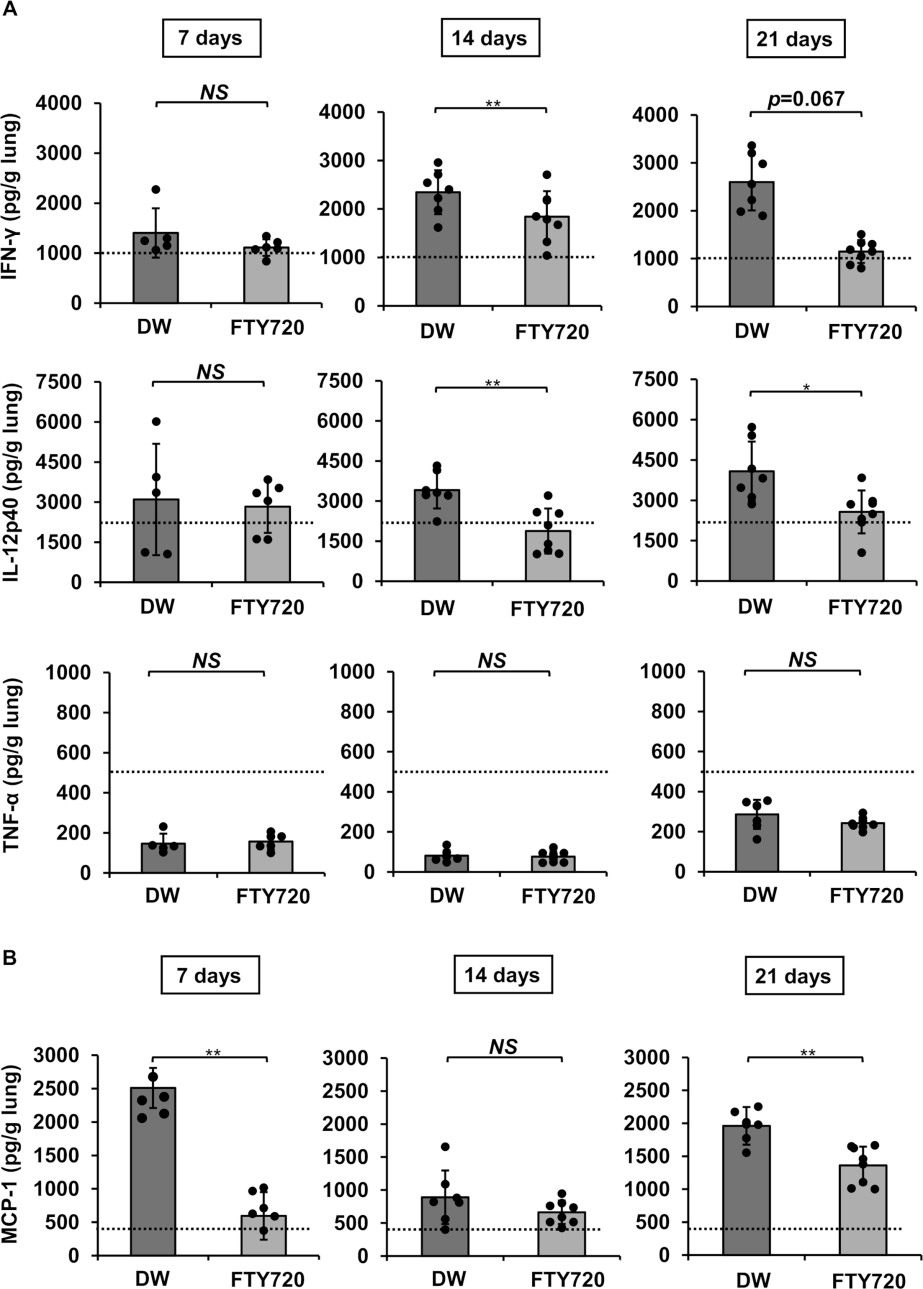
Th1-related cytokine and MCP-1 levels according to the period of FTY720 treatment. Mice with latent cryptococcal infection were orally administered FTY720 daily, with DW administration serving as the control. (**A**) IFN-γ, IL-12p40, and TNF-α in lung homogenate supernatants were measured at 7, 14, and 21 days post-treatment (*n* = 5–8 in each group). Dashed lines indicate cytokine production levels in uninfected mice. (**B**) MCP-1 levels in lung homogenate supernatants were measured at 7, 14, and 21 days post-treatment (*n* = 5–8 in each group). Dashed lines indicate the detection limit. Each symbol represents a separate mouse, and the bars indicate the mean ± SD. NS, not significant. *P < 0.05. **P < 0.01. Statistical analysis was performed using Welch’s *t*-test.

Reactivation induced by FTY720 was detected at 28 days post-treatment, suggesting that the Th1 response may have been reduced at an earlier point. We measured Th1-related cytokines in lung homogenates at 7, 14, and 21 days post-treatment (Fig. 3A). At 14 days post-treatment, IFN-γ and IL-12p40 were significantly decreased in the FTY720-treated mice compared to controls, while TNF-α was hardly detectable in both groups. Additionally, MCP-1 levels were significantly reduced at 7 days in the FTY720-treated mice compared to controls (Fig. 3B). These findings suggest that FTY720 reduces Th1 responses and MCP-1 levels, diminishes granulomatous structures, and leads to reactivation.

### CD4^+^ Teff and Tm cell kinetics in the lungs following FTY720 treatment

**Fig 4:**
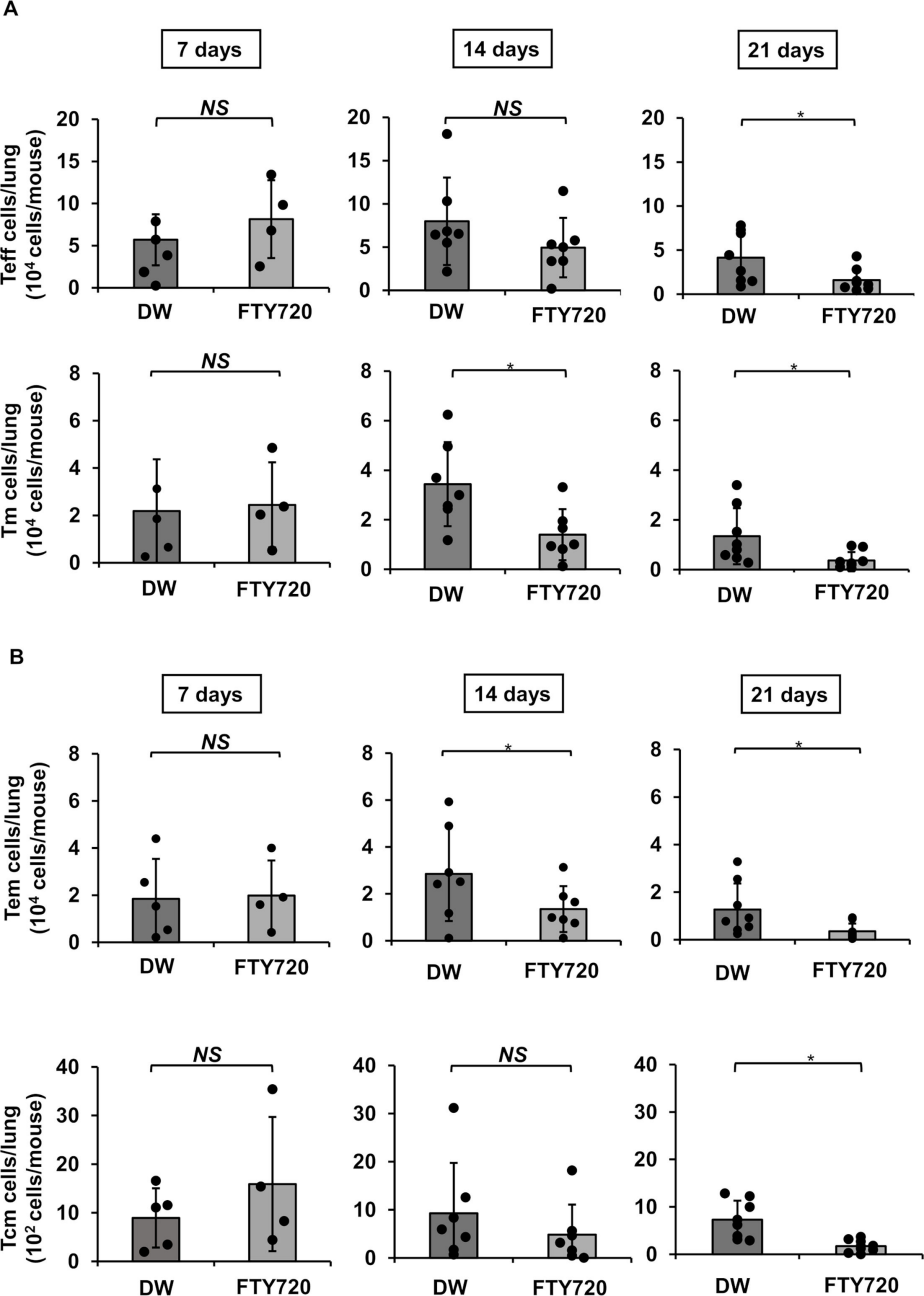
Reduction of CD4^+^ Teff and Tm cells in the lungs during FTY720 treatment. Mice with latent cryptococcal infection were orally administered FTY720 daily, with DW administration serving as the control. The number of CD4^+^ effector T cells (Teff) and Tm cells (**A**) and CD4^+^ effector Tm cells (Tem) and central Tm cells (Tcm) (**B**) in the lungs was analyzed using flow cytometry at 7, 14, and 21 days post-treatment (*n* = 5–8 in each group). Each symbol represents a separate mouse, and the bars indicate the mean ± SD. NS, not significant. *P < 0.05. Statistical analysis was performed using Welch’s *t*-test unless otherwise indicated. The Mann-Whitney U-test was used for the 21 days of Teff, Tm, and Tem, and for the 14 days of Tcm.

During primary cryptococcal infection, Th1 responses are crucial (9, 10). To identify the IFN-γ-producing cells reduced by FTY720 treatment, we analyzed CD4^+^ Teff and Tm cells in the lungs at 7, 14, and 21 days post-treatment. CD4^+^ Tm cells were significantly decreased in the FTY720-treated mice compared to controls at 14 days post-treatment, and both CD4^+^ Tm and Teff cells were significantly reduced in the FTY720-treated mice at 21 days post-treatment (Fig. 4A). Antigen-specific Tm cells differentiate into effector Tm (Tem) cells with effector functions and Tcm cells acting as reservoirs for Tem cells (29). Although CD4^+^ Tem cells were significantly decreased in FTY720-treated mice at 14 days post-treatment, Tcm cells were significantly reduced at 21 days post-treatment (Fig. 4B). This reduction in CD4^+^ Teff and Tem cells likely contributes to the decreased IFN-γ levels in the lungs, with the concurrent decrease in CD4^+^ Tcm and Teff cells suggesting FTY720’s impact (20).

### Effect of FTY720 on IFN-γ production by CD4^+^ Teff and Tm cells

**Fig 5:**
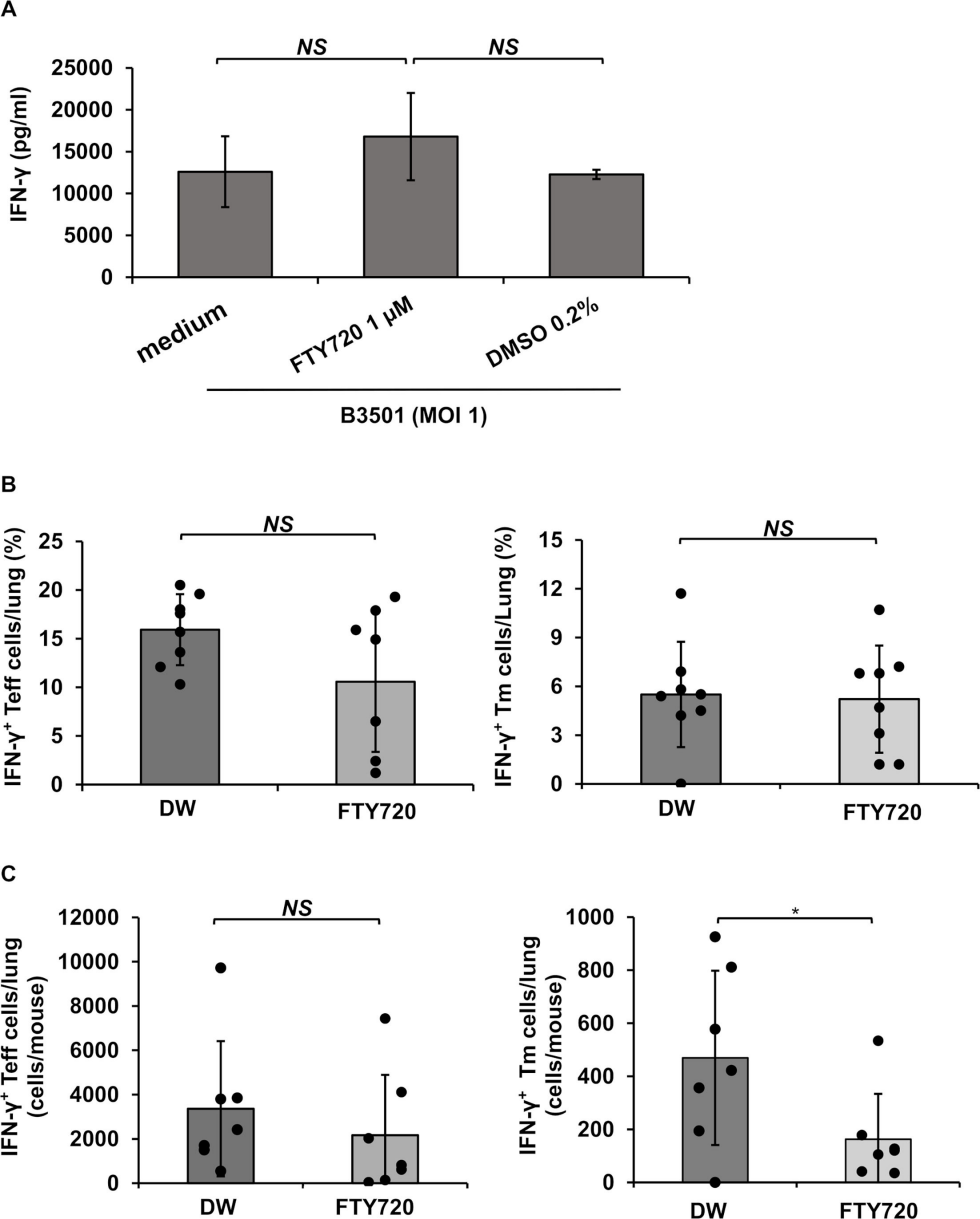
Effect of FTY720 on the production of IFN-γ by CD4^+^ Teff and Tm cells in the lungs. (**A**) Lung leukocytes (1 × 10⁶ cells/mL) from mice with latent cryptococcal infection were restimulated with *C. deneoformans* B3501 (multiplicity of infection [MOI] 1) and cocultured with the indicated dose of FTY720P or DMSO. After 48 h, supernatants were collected and IFN-γ levels were measured by enzyme-linked immunosorbent assay (ELISA) (*n* = 3 cultures in each condition). Percentages (**B**) and numbers (**C**) of IFN-γ^+^ cells in CD4^+^ Teff cells and Tm cells in the lungs were analyzed using a flow cytometer at 21 days post-treatment (*n* = 7–8 in each group). Each symbol represents a separate mouse, and the bars indicate the mean ± SD. NS, not significant. *P < 0.05. Statistical analysis was performed using Welch’s *t*-test for panel A and the Mann-Whitney U-test for panels B and C.

To determine whether the reduced IFN-γ levels in the lungs by FTY720 contribute to a decrease in IFN-γ production from CD4^+^ Teff and Tem cells or a decrease in cell number, pulmonary leukocytes from latent cryptococcal-infected mice were restimulated *ex vivo* with *C. deneoformans* and cultured in the presence of FTY720P, the biologically active phosphorylated form of FTY720. No significant difference in IFN-γ production was observed between FTY720P and dimethyl sulfoxide (DMSO) (Fig. 5A), indicating that FTY720 does not directly impair IFN-γ production. Next, we analyzed IFN-γ^+^ CD4^+^ Teff and Tm cells in the lungs at 21 days post-treatment. Although the percentage of IFN-γ^+^ cells was similar between the two groups (Fig. 5B), the number of IFN-γ^+^ CD4^+^ Tm cells was significantly lower in FTY720-treated mice compared to controls, with no significant change in IFN-γ^+^ Teff cell numbers (Fig. 5C). These results suggest that the reduced IFN-γ levels in the lungs result from a decrease in CD4^+^ Tem cells rather than impaired IFN-γ production by CD4^+^ Teff and Tm cells.

### Effect of FTY720 treatment on Cda2-specific Trm cells in the lungs

**Fig 6:**
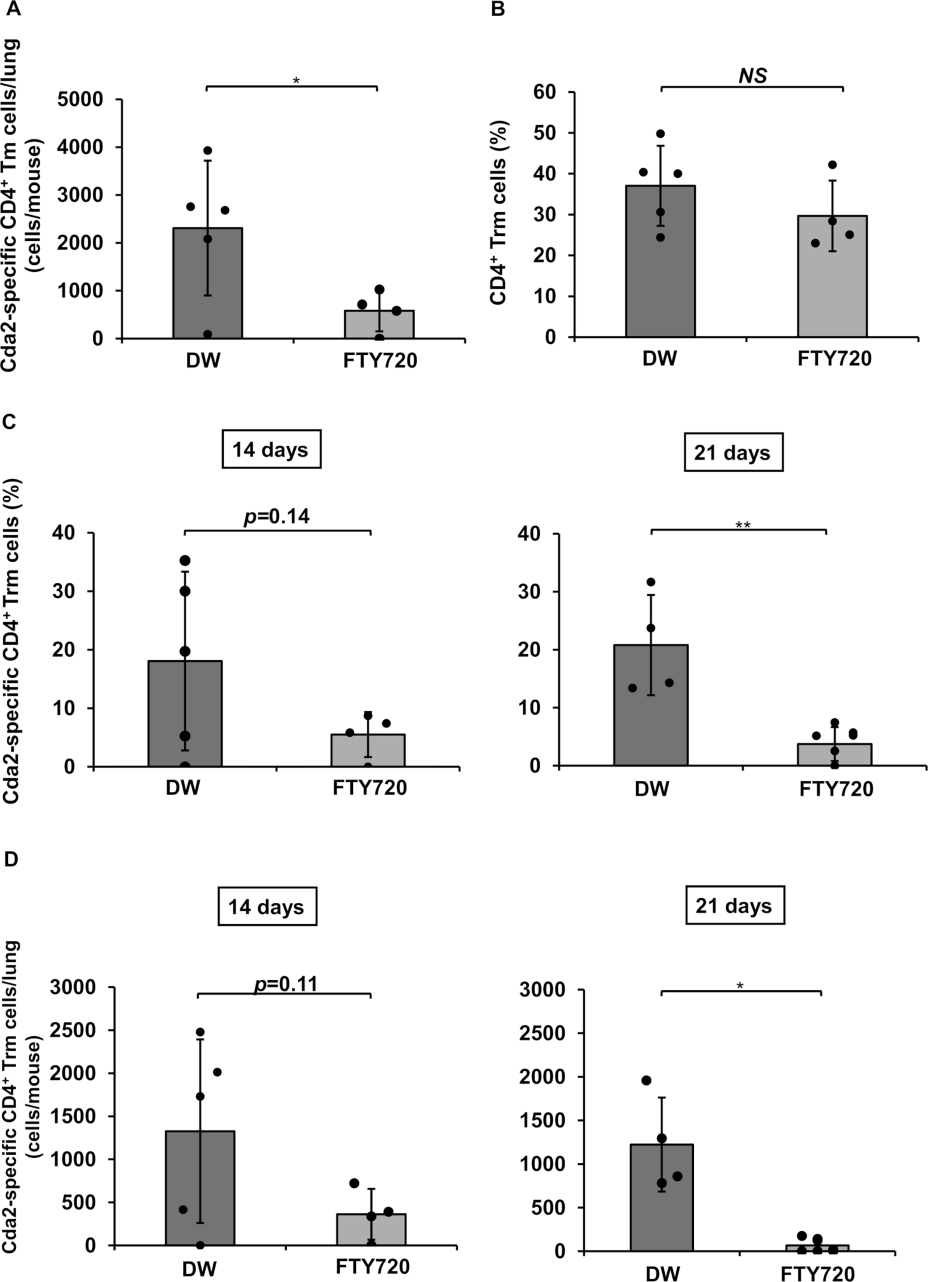
Effect of FTY720 on Cda2-specific CD4^+^ Tm and Trm cells. Mice with latent cryptococcal infection were orally administered FTY720 daily, with DW administration serving as the control. (**A**) The number of Cda2-specific CD4^+^ Tm cells in the lungs was analyzed using flow cytometry at 14 days post-treatment (*n* = 4–5 in each group). (**B**) The percentage of CD4^+^ resident Tm cells (Trm) among Tm cells in the lungs at 14 days post-treatment was analyzed using flow cytometry (*n* = 4–6 in each group). The percentage of Cda2-specific CD4^+^ Trm cells among Trm cells (**C**) and the number of Cda2-specific CD4^+^ Trm cells (**D**) in the lungs was analyzed using flow cytometry at 14 and 21 days post-treatment (*n* = 4–6 in each group). Each symbol represents a separate mouse, and the bars indicate the mean ± SD. NS, not significant. *P < 0.05. **P < 0.01. Statistical analysis was performed using Welch’s *t*-test.

To investigate whether the decrease in CD4^+^ Tem cells following FTY720 treatment was due to a reduction in Cda2-specific CD4^+^ Tm cells, we analyzed T cells using Cda2-specific MHC class II tetramer. Cda2-specific CD4^+^ Tm cells in the lungs were significantly reduced in FTY720-treated mice compared to controls at 14 days post-treatment (Fig. 6A). CD4^+^ Tcm cells, which serve as a reservoir for cryptococcal-specific Tm cells, were significantly decreased at 21 days post-treatment, following the earlier decline in CD4^+^ Tem cells (Fig. 4B). This suggests that factors beyond CD4^+^ Tcm accumulation contribute to the reduction of CD4^+^ Tem cells. Trm cells, which are present in local tissues like lungs, can rapidly activate and recruit other immune cells upon antigen encounter (30–32). These cells express CD69 and occasionally CD103. We previously reported an increase of CD4^+^ Trm cells in the lungs at primary cryptococcal infection (33), indicating their presence during latent infection. In our model, CD4^+^ Trm cells contributed approximately 30% of the Tm population (Fig. 6B). At 14 days post-treatment, the percentage and number of Cda2-specific CD4^+^ Trm cells in FTY720-treated mice were reduced compared to controls, with significant declines at 21 days (Fig. 6C and D). These results suggest that the reduction in Cda2-specific CD4^+^ Trm cells may contribute to the decreased Cda2-specific CD4^+^ Tm cells, alongside the reduction in CD4^+^ Tcm cells.

### Effect of FTY720 on CD4^+^ Tnaïve cell proliferation, CD4^+^ Teff, and Tm cell differentiation

**Fig 7:**
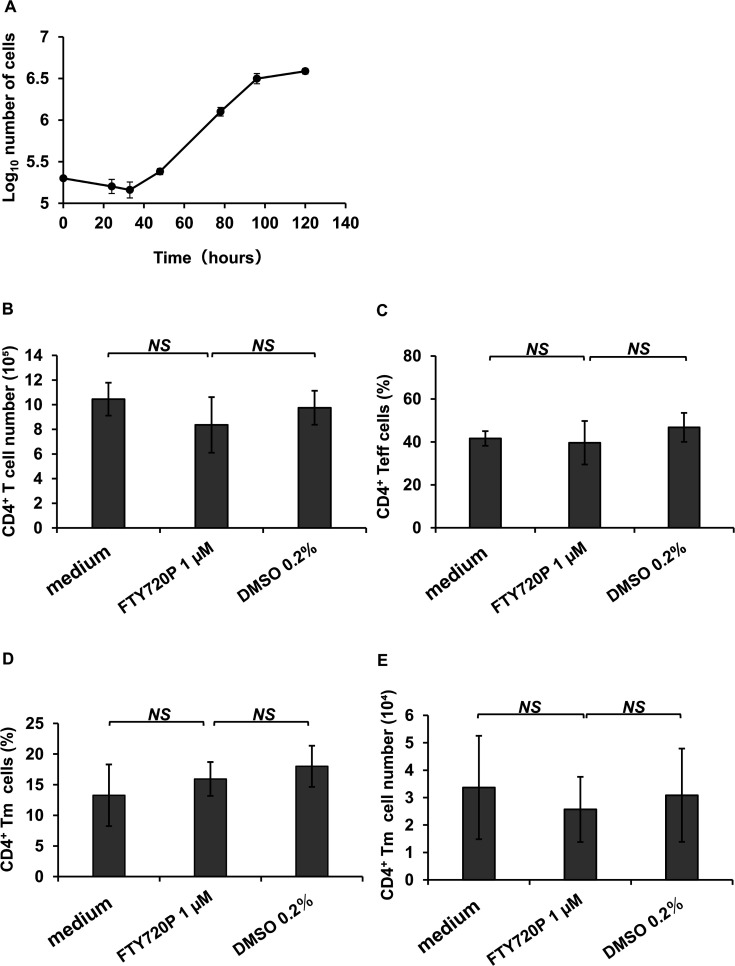
Effect of FTY720 on the proliferation of CD4^+^ Tnaïve cells and the differentiation of CD4^+^ Teff and Tm cells. CD4^+^ Tnaïve cells were isolated from splenocytes of uninfected CnT-II mice, and Teff and Tm cells were induced *in vitro* using anti-CD3, anti-CD28, anti-IL-4 mAb, rIL-2, and rIL-12p70. (**A**) Cells were counted over time to create a growth curve. (**B**) Indicated doses of FTY720P or DMSO were added at the start, and cell numbers were counted after 72 h of culture. (**C**) After 48 h of culture, the indicated concentrations of FTY720P or DMSO were added, and cells were cultured for a total of 120 h. At the end of the 120 h, cells were harvested, and the percentage of Teff cells among CD4^+^ T cells was analyzed. (**D and E**) Cells at 120 h of culture in the Teff cell generation method were washed and adjusted to a concentration of 1 × 10^6^ cells/mL. They were then cultured for 3 days in a cytokine-free medium supplemented with the indicated doses of FTY720P or DMSO. The percentage of Tm cells among CD4^+^ T cells (**D**) and the number of CD4^+^ Tm cells (**E**) were analyzed (*n* = 3 cultures in each condition). Each column represents the mean ± SD. NS, not significant. Statistical analysis was performed using Welch’s *t*-test.

To determine if FTY720 affects CD4^+^ Tnaïve cell proliferation and differentiation into Teff cells, we analyzed its impact on the *in vitro* differentiation process. First, we established a growth curve for the Teff differentiation system (Fig. 7A). The logarithmic growth phase, where cells are proliferating rapidly, is followed by a stationary phase as they reach sufficient numbers. CD4^+^ Tnaïve cells undergo proliferation and mature into Teff cells during the logarithmic growth phase (30–100 h), resulting in approximately 40% Teff cells, defined as IFN-γ^+^ cells, after 120 h of culture (Fig. S1A). We evaluated the effect of FTY720P on cell proliferation after 72 h of culture, approximately the midpoint of the logarithmic growth phase, and observed no significant difference in cell numbers between FTY720P and DMSO (Fig. 7B). Adding FTY720P between 48 h and 120 h also did not significantly alter Teff cell differentiation in FTY720P compared to DMSO (Fig. 7C). Additionally, we investigated whether FTY720P influences the differentiation of CD4^+^ Teff cells into Tm cells. Using the method described by Swain et al. (34), approximately 25% of the cells differentiated into Tm cells (Fig. S1B), and FTY720P, added at 120 h after the start of culture, did not affect Tm cell differentiation or proliferation (Fig. 7D and E). These findings suggest that the reduction in CD4^+^ Teff and Tm cells observed in FTY720-treated mice is unlikely to be due to impaired CD4^+^ T cell differentiation.

### Effects of FTY720 on cytokine production by dendritic cells

**Fig 8:**
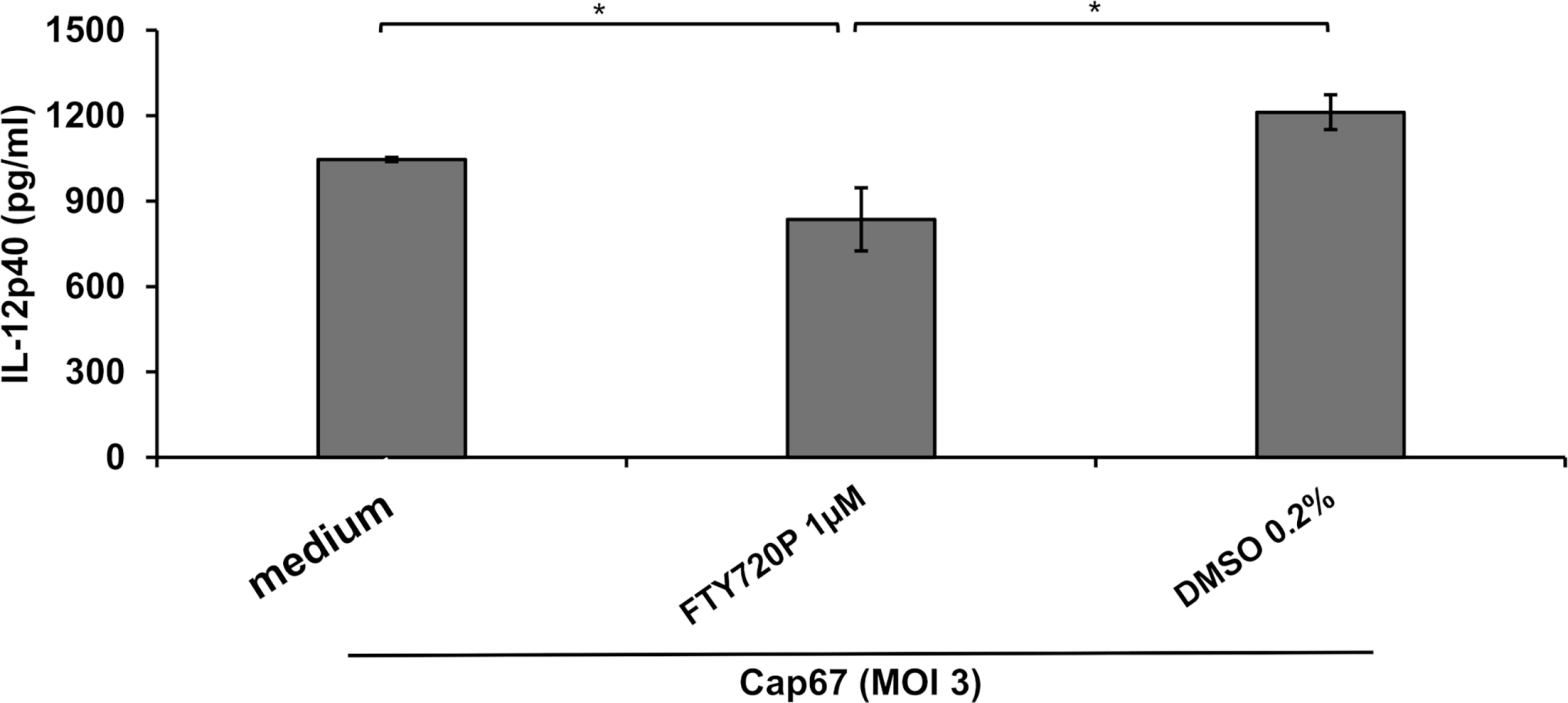
Effect of FTY720 on the cytokine production of BM-DCs. BM-DCs (1 × 10^5^ cells/mL) were stimulated with Cap67 (MOI 3) and cultured with the indicated doses of FTY720P or DMSO at 37°C with 5% CO₂ for 24 h. After 48 h, supernatants were collected, and IL-12p40 levels were measured by ELISA (*n* = 3 cultures in each condition). Each column indicates the mean ± SD. NS, not significant. *P < 0.05. Statistical analysis was performed using Welch’s *t*-test.

IL-12p40 levels in lung homogenate supernatants were lower in FTY720-treated mice compared to controls, suggesting that FTY720 affects both T cells and APCs. To investigate its impact on dendritic cell activation, bone marrow-derived dendritic cells (BM-DCs) were stimulated with Cap67 and cultured in the presence of FTY720P. Cap67 was used to avoid potential issues with the capsule inhibiting phagocytosis, as stimulation with the encapsulated B3501 may evade phagocytosis. IL-12p40 production was significantly reduced in the FTY720P-treated group, with levels lower at 1 µM compared to Cap67 mono-stimulation and the DMSO control (Fig. 8). These findings indicate that FTY720 suppresses cytokine production by BM-DCs.

## DISCUSSION

In this study, we established a novel mouse model of latent cryptococcal infection using CnT-II mice and investigated the effect of FTY720 on immune responses. In contrast, WT mice infected with B3501 failed to establish latent cryptococcal infection because long-term survival could not be achieved (Fig. S1), indicating the substantial pathogenicity of this clinical isolate. Unlike previous models that used attenuated strains such as Δ*gcs1* (14), affecting cell wall formation, and *mar1*Δ (15), which disrupts capsule formation, our model utilizes an original, non-genetically modified strain. Previous models using low-dose encapsulated strains from AIDS patients may not accurately reflect the clinical latent state due to early dissemination to the brain (27). In contrast, our model confines the infection to the lungs for an extended period, providing a more accurate representation of latent cryptococcal infection and allowing for precise analysis of the immune and cryptococcus-specific T-cell responses.

In our FTY720-treated mice, we observed decreased levels of IFN-γ, IL-12, and MCP-1, reduced granulomatous response, and increased lung fungal burdens. Th1-related cytokines such as IFN-γ, IL-12, and TNF-α are essential for granulomatous formation in primary cryptococcal infection (14, 35–37). While their roles in maintaining granulomatous structures were not well understood, our study shows that IFN-γ and IL-12 are also important for granulomatous structures maintenance. Interestingly, our latent cryptococcal infection model exhibited low TNF-α levels even in controls. Similarly, in a latent cryptococcal infection model using the *mar1*Δ strain, TNF-α levels decreased after granuloma formation, consistent with our findings (15). In contrast, TNF-α is crucial for granuloma maintenance in *Mycobacterium tuberculosis* infections (38), with studies showing neutralizing antibodies leading to granuloma collapse (39). These findings highlight the differing roles of TNF-α in tuberculosis and cryptococcosis, aligning with clinical data showing that TNF-α inhibitors increase tuberculosis risk but are less commonly associated with cryptococcosis (25, 26). Although IFN-γ, IL-12, and MCP-1 are important for maintaining granulomatous structures, the current evidence is circumstantial. To clarify their roles, neutralizing antibodies such as anti-IL-12 should be administered in a latent cryptococcal infection model to determine whether reactivation occurs.

A latent cryptococcal infection model with Δ*gcs1* suggested an important role for MCP-1 in maintaining granulomas (40). Due to the low levels of TNF-α, we measured MCP-1 as a factor involved in immune cell recruitment. In this study, MCP-1 levels in the lungs of the control group remained high but decreased in FTY720-treated mice at 7 days post-treatment. This suggests that MCP-1, rather than TNF-α, may be essential for maintaining granulomas in latent cryptococcal infection through the recruitment of immune cells. Del Poeta et al. reported that nuclear factor-κB can be activated by the Sphk1-S1P pathway, regulating MCP-1 production (40). FTY720 inhibits Sphk1, as shown in a mouse model of inflammatory bowel disease (41), and may similarly affect this pathway in the lungs. Although monocytes/macrophages, fibroblasts, and endothelial cells are known to be the main producers of MCP-1 (42), immunohistochemical analysis suggests that T and B cells may also produce MCP-1 in our model. Given that S1PR is expressed in these cells (43), FTY720 may directly influence MCP-1 production. However, our study did not analyze B cells, and detailed intracellular staining of MCP-1-expressing T cells was not performed, necessitating further investigation.

In the lungs of FTY720-treated mice, we observed a reduction in CD4^+^ Tem cells at 14 days post-treatment, followed by a decrease in CD4^+^ Teff cells at 21 days. Analysis of lung leukocytes at 21 days post-treatment revealed a decrease in IFN-γ^+^ CD4^+^ Tm cells in the FTY720-treated mice, while IFN-γ^+^ CD4^+^ Teff cells remained unchanged. FTY720 did not affect IFN-γ production in either cell type, suggesting that the reduction of IFN-γ in the lungs may result from the decrease in CD4^+^ Tem cells. This lack of effect on IFN-γ production has been previously reported in studies using blood samples from both healthy individuals and MS patients (44, 45). Tm cells are divided into Tcm and Tem cells, with Tem cells responding rapidly to specific antigens and Tcm cells serving as a reservoir with high proliferative capacity (29). We observed a decrease in CD4^+^ Tcm cells in the FTY720-treated mice at 21 days post-treatment, coinciding with the decline in CD4^+^ Teff cells, likely due to FTY720’s inhibition of T cells from secondary lymphoid organs (20).

We analyzed the effect of FTY720 on the differentiation of CD4^+^ Tm cells from Teff cells but found no significant impact. Thus, we focused on Trm cells, which are known to be induced early in cryptococcal infection (33). The role of Trm phenotype cells in primary cryptococcal infection and their immune response during latency remains unclear. In our study, around 30% of Tm cells in the lungs of mice with latent cryptococcal infection exhibited the Trm phenotype, with approximately 90% being Cda2-nonspecific. These cells may recognize other cryptococcal antigens, or Trm phenotype cells from primary infection may persist and proliferate. In FTY720-treated mice, both the percentage and number of Cda2-specific CD4^+^ Trm cells decreased. This reduction, along with a decline in Tcm cells, may explain the overall decrease in Cda2-specific CD4^+^ Tm cells. FTY720 may promote Trm cell exhaustion via the PD-1/PD-L1 pathway or impair APC function, contributing to the decline in antigen-specific Trm cells. During chronic antigen exposure, antigen-specific CD8^+^ Trm cells express PD-1 (46), which may also occur in Cda2-specific CD4^+^ Trm cells, leading to PD-1/PD-L1-mediated cell death. Future studies will explore antigen presentation and the PD-1/PD-L1 pathway. Additionally, IL-12 can induce bystander activation in CD8^+^ Trm cells and possibly in CD4^+^ Trm cells (47). The reduction in IL-12 observed at 14 days post-treatment may contribute to the decline in Cda2-specific CD4^+^ Trm cells, reducing the recruitment of Cda2-specific CD4^+^ Tem cells from the periphery and further decreasing Tm cell numbers (31).

**Fig 9:**
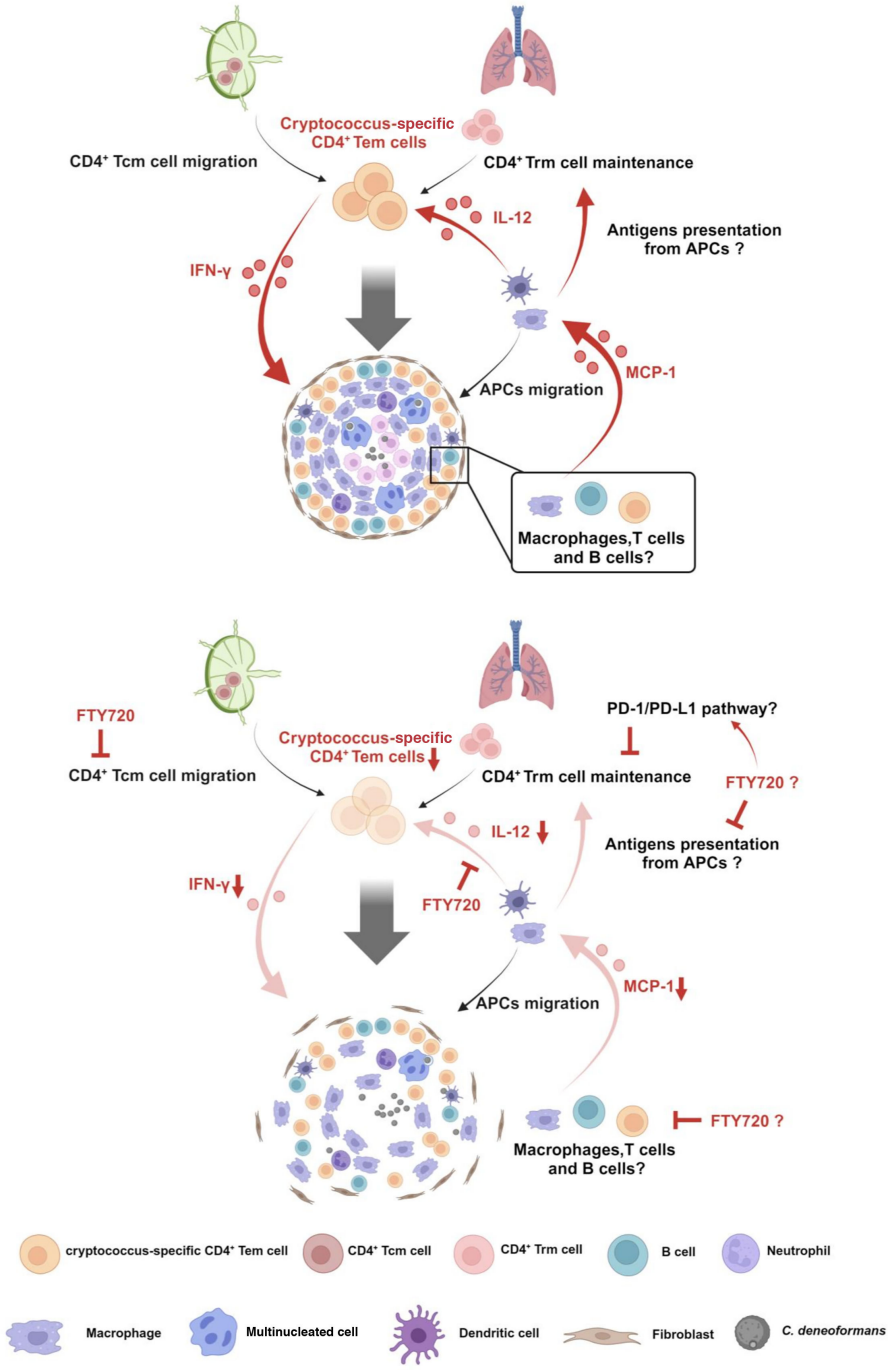
Proposed mechanism of cryptococcosis onset induced by FTY720. This figure summarizes the proposed immune mechanism involved in the latent phase of cryptococcal infection and its reactivation by FTY720. During latent infection, CD4^+^ Tem cells maintain granulomatous structures. FTY720 inhibits the migration of CD4+ Tcm cells and disrupts the maintenance of CD4^+^ Trm cells, leading to a reduction in CD4^+^ Tem cells in the lungs. This decrease lowers IFN-γ levels, which impairs granulomatous structure maintenance and promotes cryptococcal reactivation. FTY720 also impacts APCs by reducing the populations of monocytes, macrophages, and dendritic cells (48), while suppressing IL-12 production by dendritic cells, leading to reduced Th1 responses.

Based on our findings, we propose a mechanism for cryptococcosis reactivation (Fig. 9). During latent infection, CD4^+^ Tem cells play a key role in granulomatous structure maintenance. FTY720 inhibits the migration of CD4^+^ Tcm cells and disrupts the maintenance of CD4^+^ Trm cells, leading to a reduction in CD4^+^ Tem cells in the lungs. This decrease lowers IFN-γ levels, impairing granulomatous structure stability and promoting reactivation of cryptococcal infection. The reduction in Tcm cells may be a direct effect of FTY720, which also likely impacts APCs expressing multiple S1PRs. The decline in Trm cells could result from reduced antigen presentation by APCs or activation of the PD-1/PD-L1 pathway. Our study showed that FTY720 suppresses IL-12 production by dendritic cells, weakening the Th1 response, while reduced MCP-1 levels may lower the number of monocytes, macrophages, and dendritic cells in the lungs of FTY720-treated mice (48). These effects likely contribute to cryptococcal reactivation. Further analysis is needed to clarify FTY720’s effects on APCs and S1PR expression.

## MATERIALS AND METHODS

### Mice

C57BL/6 mice, purchased from CLEA (Tokyo, Japan), were used as WT. The establishment of CnT-II mice has been described previously (19). Male and female mice aged 6 to 17 weeks and weighing 16 g to 32 g were used in the experiments. The conditions of the breeding room were as previously detailed (19, 33).

### C. neoformans

*C. deneoformans* B3501 strain (kindly provided by Dr. Kwon-Chung, National Institute of Health, Bethesda, MD, USA) and its capsule-deficient strain, Cap67 (kindly provided by Dr. S. M. Levitz, University of Massachusetts Medical School, Worcester, MA, USA), were used. *C. deneoformans* B3501 and Cap67 were cultured on potato dextrose agar (PDA; Eiken, Tokyo, Japan) plates for 2 to 3 days prior to use.

### Inoculation with *C. deneoformans*

Mice were anesthetized by an intraperitoneal injection of 15 mg/kg pentobarbital (Kyoritsu Seiyaku Co., Tokyo, Japan), along with an intramuscular injection of 0.3 mg/kg midazolam (Maruishi Pharmaceutical, Osaka, Japan), and 0.02 mg/kg medetomidine hydrochloride (Nippon Zenyaku Kogyo, Fukushima, Japan). Live B3501 (1 × 10^6^ cells) were inoculated into the trachea of each mouse using a 24-gauge catheter (Terumo, Tokyo, Japan) in a volume of 50 µL.

### Treatment with FTY720

FTY720 (Cayman, Ann Arbor, MI, USA) was dissolved in DW (Fuso Pharmaceutical Industries, Osaka, Japan) to a concentration of 10.5 μg/mL, and mice were orally administered FTY720 ad libitum every day from 2 to 3 months post-infection (49). Control mice received DW orally on the same schedule.

### Histological examination

Lung specimens were fixed in 10% neutral buffered formalin, dehydrated, and embedded in paraffin. Sections were cut at 5 μm intervals and stained with HE, PAS, and immunostaining at the Biomedical Research Core, Animal Pathology Platform, Tohoku University School of Medicine. Details of the immunohistochemistry protocol are presented in Table 1. Observations were made using a LEICA DM750 (Leica Microsystems, Wetzlar, Germany), and photographs were taken with a LEICA DMC2900 (Leica Microsystems). The granulomatous response in 12 HE-stained sections from each group was measured with ImageJ software (https://imagej.net/ij/), and the percentage relative to the total lung area was calculated.

**TABLE 1:** Details of the immunohistochemistry protocol^*a*^

Target protein	Antigen retrieval	Blocking reagent	Primary Ab clone/origin, labeling	Endogenous peroxidase removal	Secondary Ab labeling	Substrate reagent	Counterstaining reagent
CD3	Histofine solution (pH 9, Nichirei), 120°C, 5 min	None	Clone 413591/ Rabbit (Nichirei, Tokyo, Japan), used undiluted, incubated overnight at 4°C	Methanol and 30% H_2_O_2_	Histofine Simple Stain Mouse MAX-PO (R, Nichirei)	3,3′ Diaminobenzidine (DAB; Wako, Osaka, Japan)	Hematoxylin (Merck Millipore, Burlington, MA, USA)
B220	0.01M citrate buffer (pH 6), 120°C, 5 min	Protein Block Serum-Free (Code X0909, Dako)	Clone RA3-6B2/Rat (BD Biosciences), incubated for 90 min at room temperature, diluted 1:200, followed by streptavidin		None		
F4/80	Target Retrieval Solution (pH 6, Dako, Carpinteria, CA, USA), 120°C, 5 min	None	Clone 123101/Rat (BioLegend), incubated overnight at 4°C, diluted 1:100		Histofine Simple Stain Mouse MAX-PO (Rat, Nichirei)		
MCP-1	0.01M citrate buffer (pH 6), 120°C, 5 min	Histofine Mouse Stain kit (Nichirei)	Clone 2D8/Mouse (Novus, Littleton, CO, USA), incubated overnight at 4°C, diluted 1:200		Histofine Simple Stain Mouse MAX-PO (Mouse, Nichirei)		

^*a*^ Reagents and methods used for immunostaining are shown.

### Enumeration of viable *C. deneoformans*

Mice were sacrificed, and their lungs were carefully dissected, excised, and homogenized separately in 2 mL of DW using a stainless-steel mesh at room temperature. The homogenates were then appropriately diluted with DW, and 100 µL of each diluted sample was inoculated on PDA plates. The plates were incubated for 2 to 3 days, after which the resulting colonies were counted (18, 19).

### Preparation of lung homogenate supernatant

Mice were sacrificed, and the lungs were removed and homogenized in 2 mL of phosphate-buffered saline (PBS) using a stainless-steel mesh at room temperature. The homogenates were centrifuged, and the supernatants were filtered through a 0.2 µm sterilizing filter (Sartorius, Göttingen, Germany) and stored at −80°C (19).

### Preparation of lung leukocytes and stimulation of lung leukocytes with B3501

Pulmonary leukocytes were prepared as previously studied (18, 19, 33). Briefly, after opening the chest, the lungs were perfused with 5 mL of cold saline via the right ventricle, excised, and placed in RPMI 1640 medium (Nacalai Tesque, Kyoto, Japan) supplemented with 10% fetal bovine serum (FBS), antibiotics, HEPES, 2-mercaptoethanol, collagenase, and DNase I (all from Sigma-Aldrich). The lungs were homogenized on a stainless-steel mesh and incubated at 37°C for 60 min. The resulting cell suspension was filtered through a 40 µm strainer (BD Biosciences), centrifuged, and resuspended in 40% Percoll, then layered on 80% Percoll (Pharmacia, Uppsala, Sweden). Following centrifugation at 600 × *g* for 20 min at 20°C, the interlayer was collected, and red blood cells were lysed with hemolysis buffer (155 mmol/L NH_4_Cl, 10 mmol/L KHCO_3_, 0.1 mmol/L EDTA, pH 7.4). The cells were washed three times with RPMI 1640 medium or staining buffer (PBS with 1% FBS and 0.1% sodium azide) and then counted using a hemocytometer. Lung leukocytes (1 × 10^6^ cells/mL) were stimulated with B3501 at an MOI of 1 and cultured with FTY720P (Cayman), dissolved in DMSO, at a final concentration of 1 µM, or DMSO (Sigma-Aldrich) at 0.2% as a control for 48 h at 37°C with 5% CO_2_. Concentrations of DMSO above 0.6% were toxic to immune cells (data not shown). Supernatants were stored at −80°C until analysis. Although antifungal activity was observed at FTY720P 10 µM, DMSO at the same concentration (2%) had similar effects, suggesting that the antifungal activity might be due to DMSO (Table S1). At the concentrations used, neither FTY720P nor DMSO exhibited antifungal activity.

### Flow cytometry

Cells were washed with staining buffer (PBS with 1% FBS [Biowest, Nuayer, France]) and 0.1% sodium azide and incubated with anti-FcγRII/III mAb (clone 2.4G2, Kirkegaard & Perry Laboratories) on ice for 15 min. Surface staining was performed using APC-CD3 (clone 17A2), PE-CD4 (clone RM4-5), Pacific Blue-CD44 (clone IM7), and Biotin-CD127 (clone A7R34) mAbs (all BioLegend, San Diego, CA, USA). For effector and central Tm (Tem and Tcm) cell analysis, PE/Cy7-CD62L mAb (clone MEL-14, BioLegend) was included. After 30 min, cells were stained with PerCP/Cy5.5-Streptavidin (BioLegend), washed, and dead cells were identified using a Fixable Aqua Dead Cell Stain Kit (Invitrogen, Carlsbad, CA, USA). To analyze IFN-γ expression, cells were stimulated with ionomycin (2 µg/mL), PMA (20 ng/mL), and monensin (8 µM) (all Sigma-Aldrich, St. Louis, MO, USA) for 4 h at 37°C, 5% CO_2_. After the surface and dead cell staining, cells were fixed with 4% paraformaldehyde/PBS, permeabilized with Perm/Wash buffer (BD Biosciences), and stained with FITC-IFN-γ (clone XMG1.2, BioLegend). For Cda2-specific CD4^+^ Tm and resident-memory T cells (Trm) analysis, cells were incubated with PE-MHC-Class II Tetramer (Cda2 peptide, ProImmune, Oxford, UK) at 37°C for 2 h, followed by staining with APC-CD3, APC/Cy7-CD4 (clone GK1.5), Pacific Blue-CD44, Biotin-CD127, FITC-CD103 (clone 2E7), and PE/Cy7-CD69 (clone H1.2F3) mAbs (all BioLegend). Cells were analyzed using a BD FACS Canto II flow cytometer (BD Biosciences) (Fig. S2).

### Generation of CD4^+^ Teff and Tm cells

CD4^+^ Tnaïve cells were isolated from the spleens of unimmunized CnT-II mice as previously described (20). A 96-well flat-bottom plate was coated with anti-mouse CD3 mAb (5 µg/mL, clone 17A2, eBioscience, San Diego, CA, USA) and incubated at 37°C with 5% CO_2_ for 2 h. Tnaïve cells (1 × 10⁶/mL) were cultured with anti-mouse CD28 mAb (2 µg/mL, clone 37.51, eBioscience), anti-mouse IL-4 mAb (10 µg/mL, clone 11B11, BioLegend), recombinant IL-12p70 (10 ng/mL, PeproTech, Cranbury, NJ, USA), and recombinant IL-2 (20 ng/mL, PeproTech) at 37°C with 5% CO_2_. After 48 h, the culture was scaled up to a 24-well plate and continued with anti-mouse IL-4 mAb (10 µg/mL), recombinant IL-12p70 (10 ng/mL), and recombinant IL-2 (20 ng/mL) for an additional 72 h to generate Teff cells (29). CD4^+^ Teff cells were washed three times with RPMI 1640 medium and cultured at 37°C with 5% CO₂ for 72 h to generate CD4^+^ Tm cells (34). FTY720P (1 µM) or DMSO (0.2%) was added during Teff cell generation and at 48 h for further analysis. After 72 h, cell counts were assessed for proliferation, and IFN-γ expression in CD4^+^ Teff cells was analyzed by flow cytometry to evaluate differentiation. CD4^+^ Tm cell differentiation was examined by culturing CD4^+^ Teff cells with FTY720P (1 µM) or DMSO (0.2%) for 72 h, with a percentage of Tm cells calculated.

### Preparation and culture of dendritic cells

BM-DCs were prepared following previously described methods (19). BM-DCs (1 × 10⁵ cells/mL) were then cultured with Cap67 (MOI of 3), FTY720P (1 µM), or DMSO (0.2%) at 37°C with 5% CO_2_ for 24 h. The supernatant was collected and stored at −80°C until use. The effect of FTY720P on Cap67 was similar to that on B3501, and no effects were observed at the concentrations used (Table S1).

### Cytokine assay

The concentrations of IFN-γ, IL-12p40, TNF-α, and MCP-1 were measured using an ELISA kit (BioLegend for IFN-γ, IL-12p40, and TNF-α; Novus Biologicals, Centennial, CO, USA for MCP-1).

### Statistical analysis

All statistical analyses were performed using EZR (Jichi Medical University Saitama Medical Center, Saitama, Japan), a graphical user interface for R (The R Foundation for Statistical Computing, Vienna, Austria). Data are presented as the mean ± SD. Differences between groups were examined for statistical significance. Normality was confirmed using the Shapiro-Wilk test. For normally distributed data, the Welch’s *t*-test was used; for non-normally distributed data, the Mann-Whitney U-test was applied. Analysis of lung leukocyte counts was performed using the Smirnov-Grubbs test for outliers. A *P*-value <0.05 was considered significant.

## SUPPLEMENTAL MATERIAL

Fig. S1 iai.00612-25-s0001.tif Time course of pulmonary fungal burden and survival in C57BL/6 mice following intratracheal B3501 infection.

Fig. S2 iai.00612-25-s0002.tif Pulmonary fungal burden and survival after intratracheal B3501 infection in C57BL/6 mice.

Fig. S3 iai.00612-25-s0003.tif Flow cytometric analysis of CD4+ T cell subsets with representative histograms.

Fig. S4 iai.00612-25-s0004.tif Definition of tetramer-positive CD4⁺ Tm cells and representative tetramer plots of Trm subsets.

Table S1 iai.00612-25-s0005.pdf Antifungal activity of FTY720P assessed by CLSI M27-A2 broth microdilution (50).

ASM does not own the copyrights to Supplemental Material that may be linked to, or accessed through, an article. The authors have granted ASM a non-exclusive, world-wide license to publish the Supplemental Material files. Please contact the corresponding author directly for reuse.
