# Supplementary figures and images for: Immunological mechanism behind reactivated cryptococcosis in persistently infected mice following FTY720 treatment
Source: Infect Immun. 2026 Apr 30;94(6):e00612-25. doi: 10.1128/iai.00612-25 (PMC13248744; doi:10.1128/iai.00612-25)

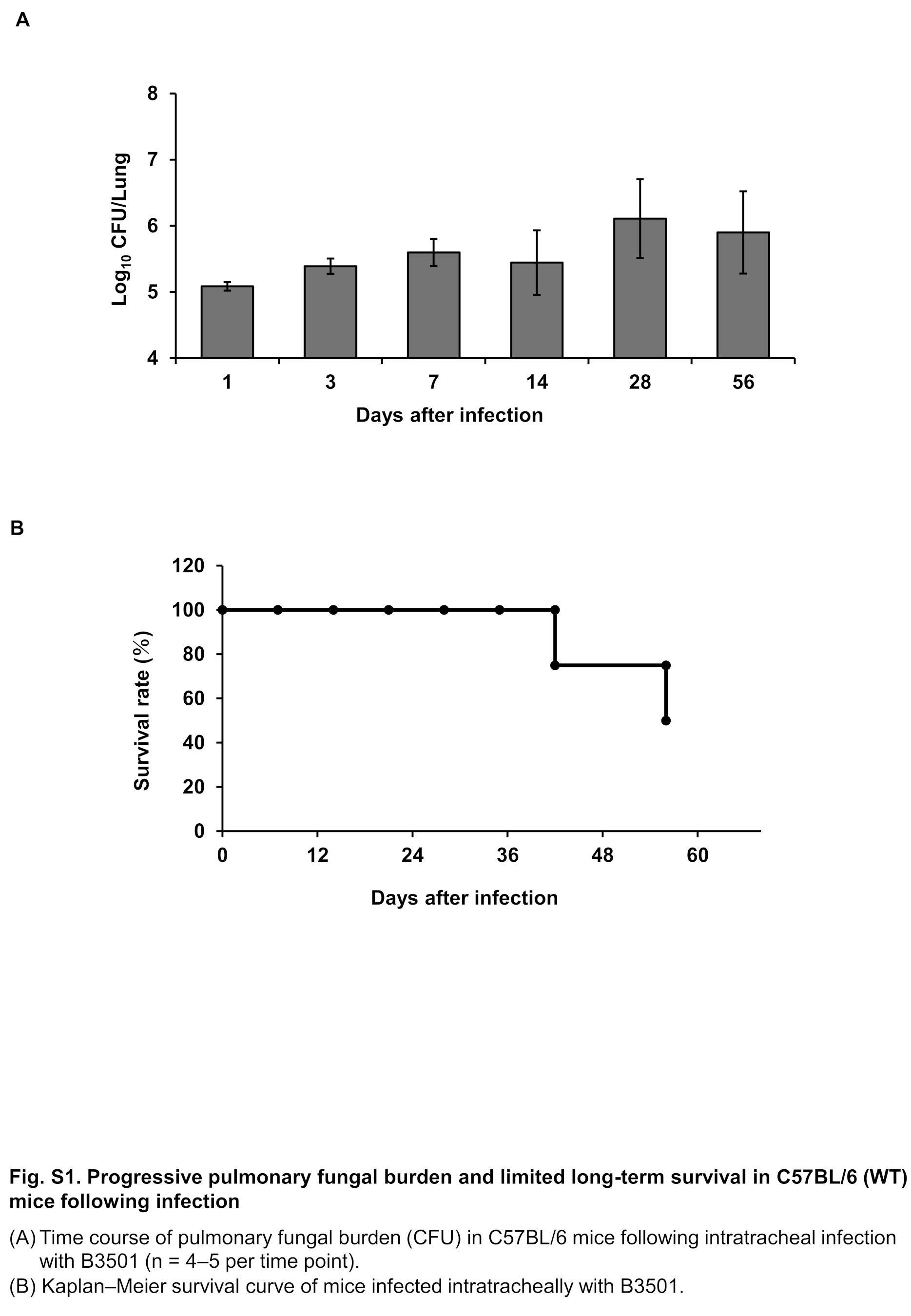

Supplement: Fig. S1 — Time course of pulmonary fungal burden and survival in C57BL/6 mice following intratracheal B3501 infection. [file iai.00612-25-s0001.tif]

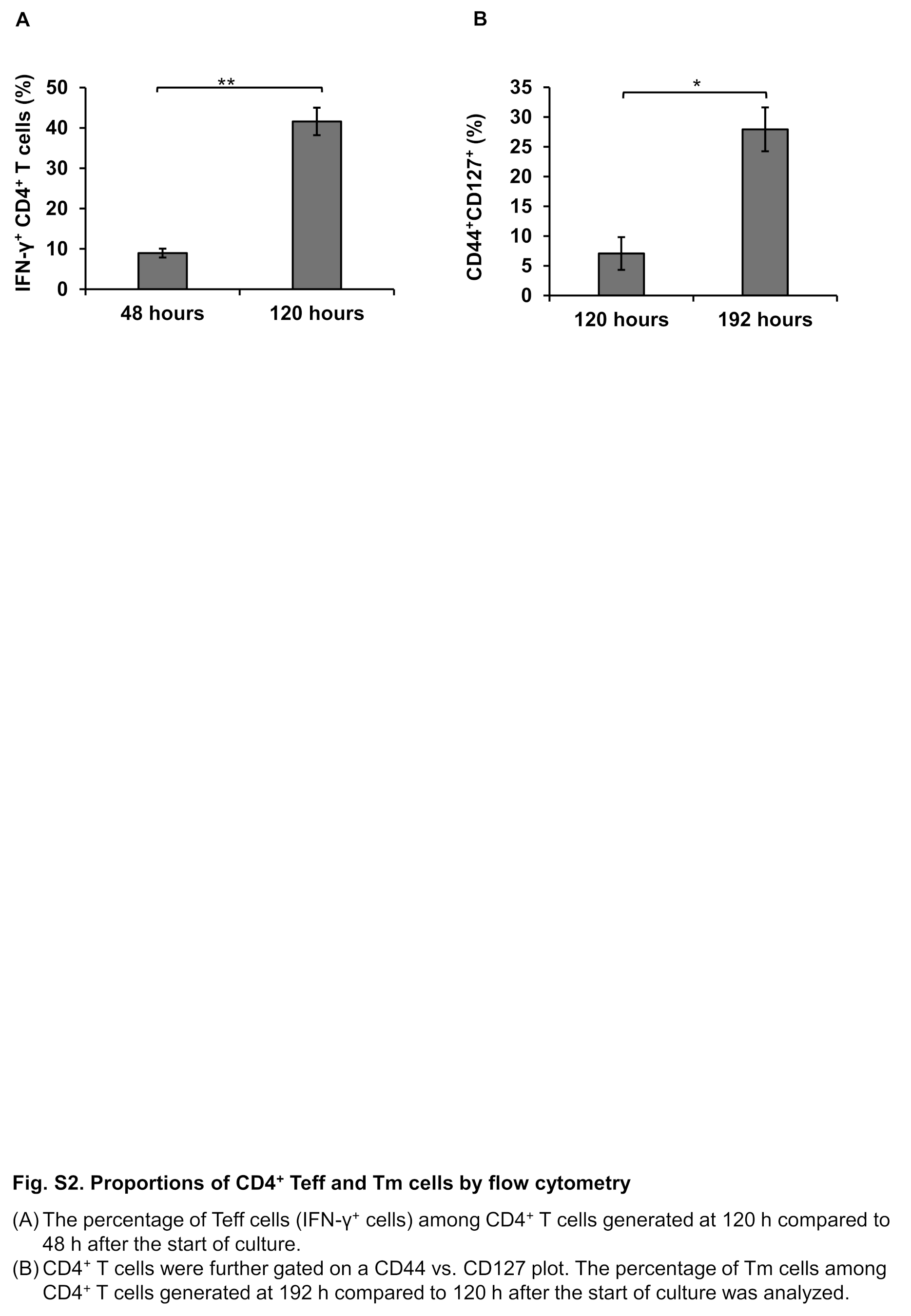

Supplement: Fig. S2 — Pulmonary fungal burden and survival after intratracheal B3501 infection in C57BL/6 mice. [file iai.00612-25-s0002.tif]

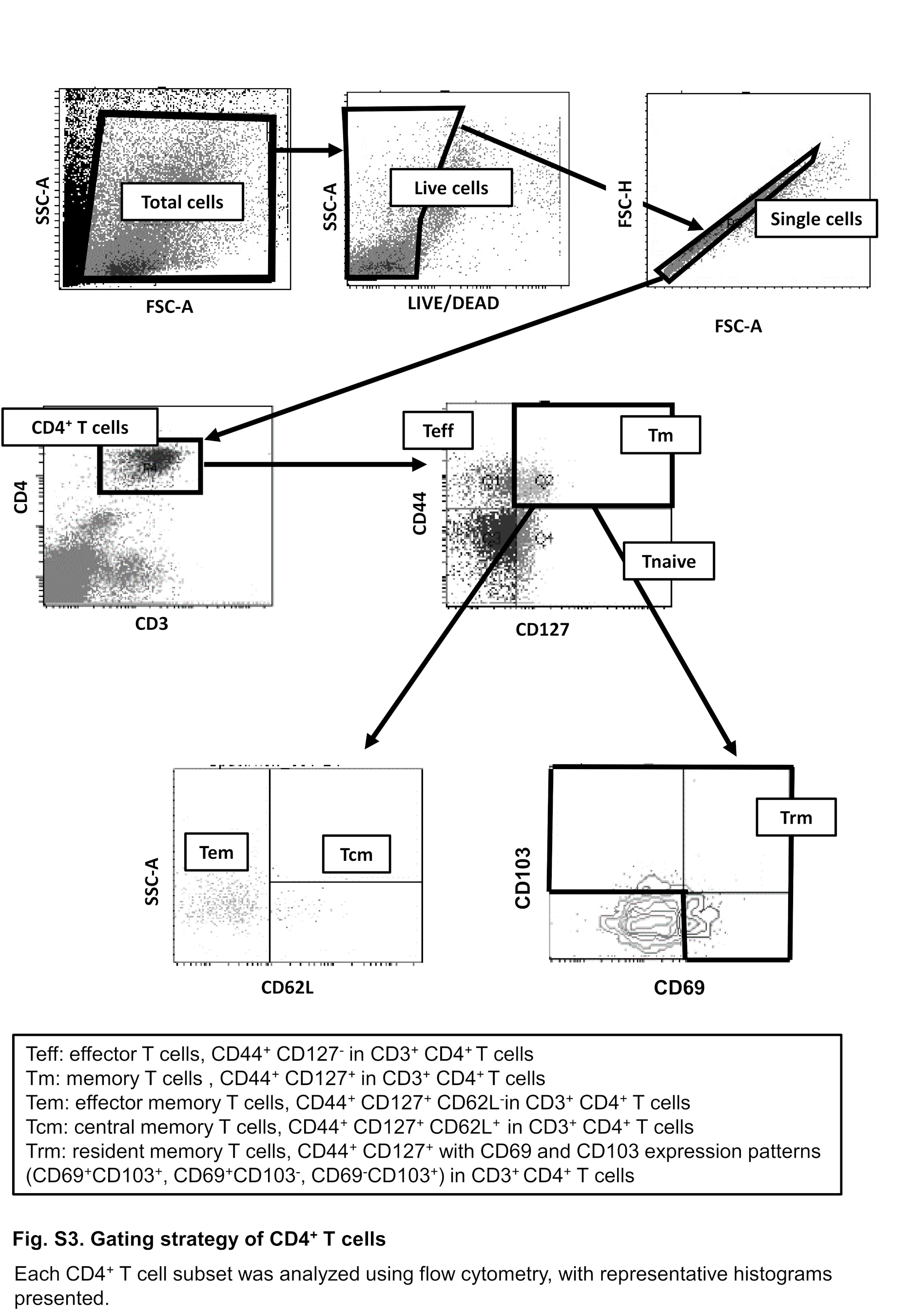

Supplement: Fig. S3 — Flow cytometric analysis of CD4+ T cell subsets with representative histograms. [file iai.00612-25-s0003.tif]

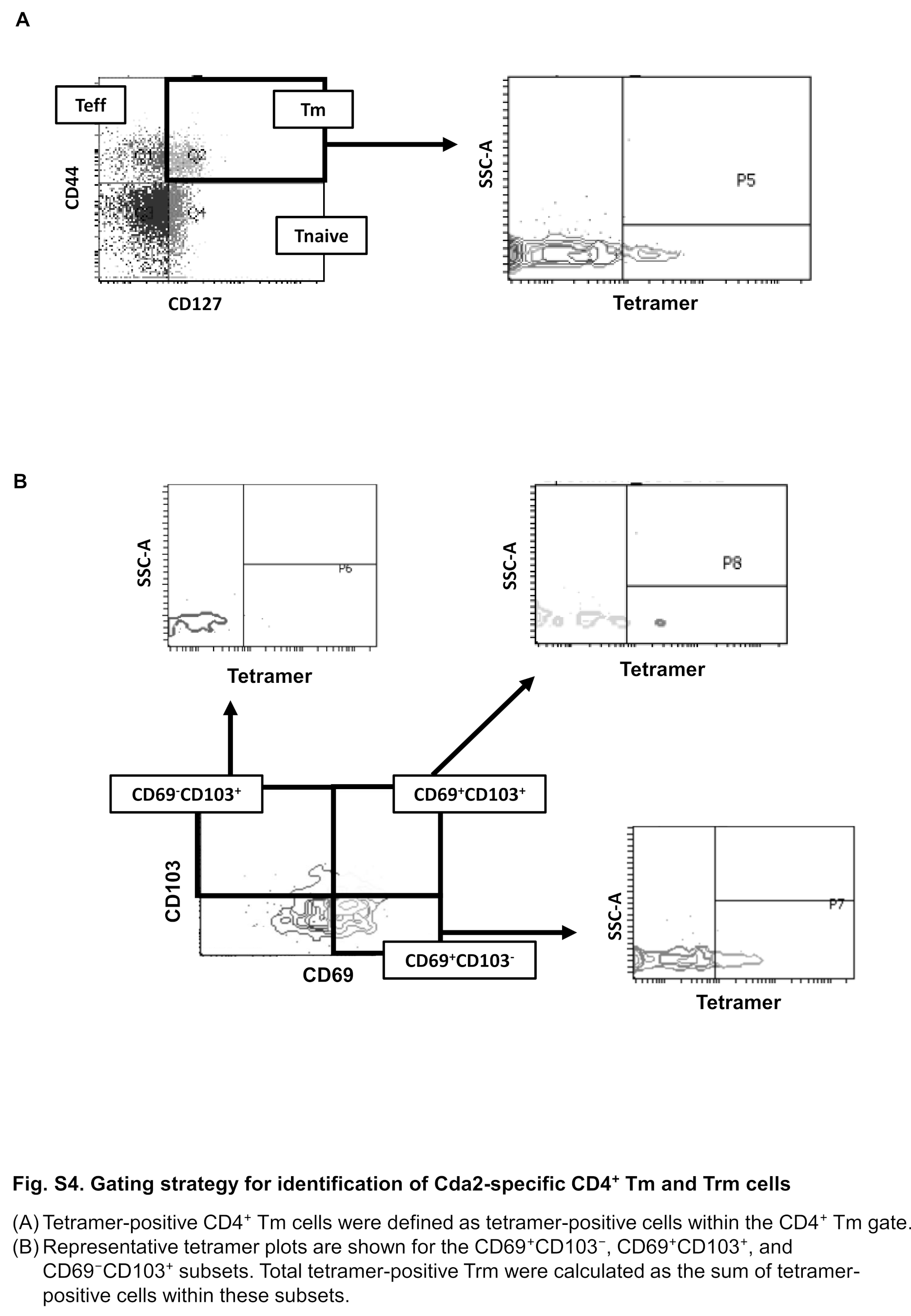

Supplement: Fig. S4 — Definition of tetramer-positive CD4⁺ Tm cells and representative tetramer plots of Trm subsets. [file iai.00612-25-s0004.tif]
