# Supplementary material for: Immunological mechanism behind reactivated cryptococcosis in persistently infected mice following FTY720 treatment
Source: Infect Immun. 2026 Apr 30;94(6):e00612-25. doi: 10.1128/iai.00612-25 (PMC13248744; doi:10.1128/iai.00612-25)
Supplement: Table S1 — Antifungal activity of FTY720P assessed by CLSI M27-A2 broth microdilution (50). [file iai.00612-25-s0005.pdf]

	B3501	Cap67
FTY720P (μM)	10	10
DMSO (%)	2	2
Amphotericin B<br>(μg/ml, Clinigen Group plc, Burton-on-Trent, UK)	0.06	0.03

Table S1. *in vitro* susceptibility testing of FTY720P

The antifungal activity of FTY720P was measured by the CLSI M27-A2 broth microdilution method (50).
